# Difference in Moxibustion-Induced Microcirculatory Responses between the Heart and Lung Meridians Assessed by Laser Doppler Flowmetry

**DOI:** 10.1155/2021/6644625

**Published:** 2021-04-01

**Authors:** Yongliang Jiang, Hantong Hu, Xiaoyu Li, Jiali Lou, Yajun Zhang, Xiaofen He, Yuanyuan Wu, Xiaomei Shao, Jianqiao Fang

**Affiliations:** ^1^Department of Neurobiology and Acupuncture Research, The Third Clinical Medical College, Zhejiang Chinese Medical University, Key Laboratory of Acupuncture and Neurology of Zhejiang Province, Hangzhou 310053, China; ^2^Department of Acupuncture and Moxibustion, The Third Affiliated Hospital of Zhejiang Chinese Medical University, Hangzhou City, Zhejiang Province, China

## Abstract

**Objective:**

By comparing the differences in microcirculatory responses of the heart and lung meridians induced by moxibustion on these two meridians, respectively, this study aimed to investigate the specificity for site-to-site association on body surface between different meridians.

**Methods:**

Eighty healthy adults were enrolled and divided into the lung meridian intervention group and heart meridian intervention group in a ratio of 1 : 1. Three-channel laser Doppler flowmetry was used to monitor microcirculatory responses for the heart and lung meridians. Primary outcome was change of blood perfusion units (PU) of three measurement sites along the two meridians.

**Results:**

In the lung meridian intervention group, following moxibustion performed at LU5 of the lung meridian, PU in the distal site of the lung meridian increased significantly. By contrast, the PU of HT3 in the heart meridian, which was nearest to the moxibustion site, did not change significantly. PU in the distal site of the heart meridian declined. Meanwhile, significant difference in PU change was detected between the distal site of the lung meridian and the other two control sites of the heart meridians during moxibustion and postmoxibustion. Alternatively, similar to the results of the lung meridian intervention group, the specificity of microcirculatory response between the heart and lung meridians was observed in the heart meridian intervention group.

**Conclusions:**

For the heart and lung meridians, the effect of moxibustion-induced microcirculatory response may be more related to meridian routes than the specific distance between two sites located at different meridians, thereby supporting possible specificity for site-to-site association on the body surface between these two meridians. Nevertheless, given that only two meridians and limited measurement sites were compared, all current findings are not sufficiently robust. Further research should be conducted to investigate more meridians and measurement sites.

## 1. Introduction

In recent years, acupuncture has received increasing attention in many western countries. Convincing evidence based on meta-analyses has proved that acupuncture is effective for treating a wide range of diseases, such as pain conditions [1, 2], respiratory diseases [3, 4], cardiovascular disorders [5, 6], and digestive diseases [7, 8]. Although acupuncture is gaining increasing acceptance, as the guidance of almost all acupuncture clinical practices for thousands of years [9], the meridian theory and existence of meridian systems have been questioned abroad [10–12].

According to the classical theories of acupuncture and traditional Chinese medicine (TCM), meridians distribute on the surface of the whole body vertically and horizontally, integrating the surface of the body with internal organs, thus transforming the whole body into one entire organ. That is to say, the essence of the meridian theory and meridian systems mainly manifests its summaries regarding the fundamental rules for correlation/specificity of different sites of the body.

In the past few years, research highlights of meridian studies have focused on the acupoint specificity [13–15] and the correlation between body surface (i.e., meridians or acupoints) and internal organs [16–18]. Apart from these highlights, specificity for site-to-site association on the body surface between different meridians is also a core scientific problem to be solved in meridian studies [19]. A clearer understanding plays an important role in comprehensively revealing the fundamental rules of meridians and underlying mechanism of the specific effect of acupoints. It is also pragmatically valuable for enhancing the therapeutic effect of acupuncture in clinical practice. However, the value of its investigation was always underestimated or ignored in previous studies.

To the best of our knowledge, the specificity for site-to-site association on the body surface between two specific meridians has not yet been systematically examined in humans. Therefore, the aim of this pilot study was to investigate the specificity for site-to-site association on the body surface between two specific meridians by using laser Doppler flowmetry (LDF). The heart and lung meridians were chosen as the two studied meridians and healthy adults were enrolled. We hypothesized that moxibustion on the stimulated meridian would induce different microcirculatory responses between the lung meridian and heart meridian. By comparison of microcirculatory response between these two meridians, the specificity for site-to-site association on the body surface between different meridians would be investigated.

## 2. Materials and Methods

### 2.1. Sample Size Estimation

This is a pilot study for a full-scale trial in the future, the protocol of which has been published [20]. It is a meridian study concerning comparison of microcirculatory characteristic between the heart and lung meridians. Compared with general clinical trials, there was no unified standard for the sample size estimation. Hence, the sample size was mainly estimated according to similar meridian studies [21] and feasibility. Finally, 80 participants were planned.

### 2.2. Subjects

A total of 80 healthy volunteers were recruited and divided into the lung intervention group (20 male and 20 female, aged 27.75 ± 3.79 years) and heart intervention group (20 male and 20 female, aged 27.93 ± 3.35 years) in a ratio of 1 : 1. Ethical approval (approval no.: ZSLL-KY-2019-001A-01) was obtained from the Ethics Committee of the Third Affiliated Hospital of Zhejiang Chinese Medical University. Informed consents were signed by all participants. All included healthy adults should provide a recent medical examination report to confirm that they had not major systemic diseases, such as cardiovascular, respiratory, digestive, urinary, hematological, endocrine, and neurological disease. They should not take any medication in the past three months. Participants who had alcohol dependence or drug abuse were excluded.

### 2.3. Experimental Procedure

To minimize the interference effect induced by confounding factors, all LDF examinations were performed in a quiet experimental room, controlled for temperature and humidity (25°C ± 1°C; relative humidity 30–40%), in the morning at about the same time of day. All subjects were refrained from consuming coffee, tea, alcohol, or smoking cigarettes on the examination day. Exercise and food were also restricted at least one hour before the experiment.

The participants were asked to stabilize for 30 mins in a supine position before LDF examination. They were informed to keep silent and breathe normally and avoid limb movement during the whole assessment period. A three-channel LDF (PeriFlux system 5000, Sweden) was used to monitor the microcirculatory flux of the heart and lung meridians, which had a wavelength of 780 nm and time constant 0.2. The fiber probe model attached to measurement sites was 407, which could detect surface microcirculatory flux. The LDF examination involved a 25 min recording of blood perfusion, including 5 min premoxibustion, 15 min moxibustion, and 5 min postmoxibustion phases. In addition, before and immediately after the LDF examination, fundamental physiological parameters of the subjects were measured using a manometer and thermometer, including heart rate, systolic blood pressure, diastolic blood pressure, and body temperature.

**Figure 1:**
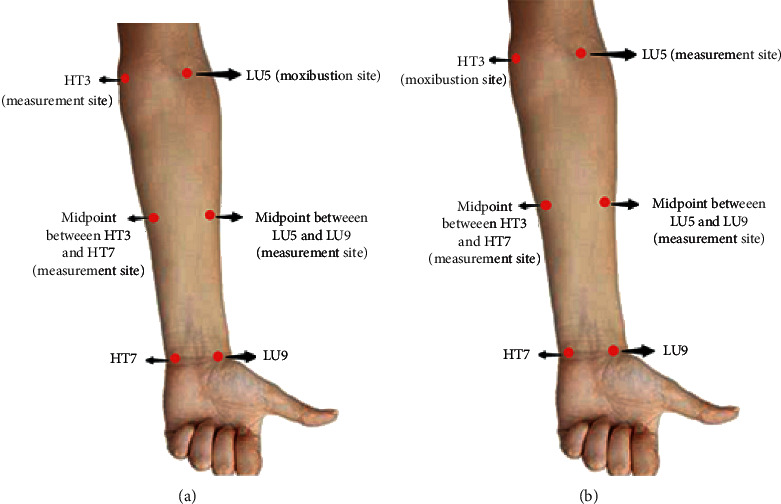
Moxibustion site and LDF measurement sites of the heart and lung meridians. (a) Moxibustion site and LDF measurement sites in the lung meridian intervention group. (b) Moxibustion site and LDF measurement sites in the heart meridian intervention group.

The intervention procedures and LDF measurement sites of the heart and lung meridians in both groups are shown in Figures 1(a) and 1(b), respectively. The anatomical locations of the acupoints and the LDF measurement sites are displayed in Table 1.

#### 2.3.1. Lung Meridian Intervention Group

A moxa stick was ignited and inserted into a homemade holder to adjust appropriate height and angle. In this group, moxibustion was performed at LU5 of the lung meridian. The fiber probes of the three-channel LDF to monitor microcirculation flux were attached in three measurement sites simultaneously, which included the midpoint of the lung meridian along the left forearm (i.e., the midpoint between LU5 and LU9), midpoint of the heart meridian along the left forearm (i.e., the midpoint between HT3 and HT7), and HT3 of the heart meridian.

#### 2.3.2. Heart Meridian Intervention Group

In this group, moxibustion was performed at HT3 of the heart meridian. Three LDF measurement sites included the midpoint of the heart meridian along the left forearm (i.e., the midpoint between HT3 and HT7), midpoint of the lung meridian along the left forearm (i.e., the midpoint between LU5 and LU9), and LU5 of the lung meridian.

### 2.4. Data Analysis

**Figure 2:**
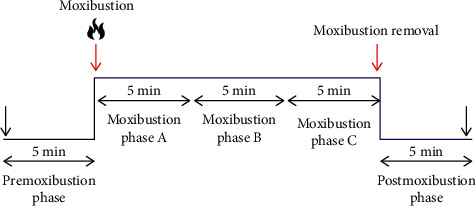
LDF measurement procedure. LDF: laser Doppler flowmetry.

All LDF flux signal processing was performed with PeriSoft (PeriFlux, Sweden), in which microcirculation flux was measured and presented as blood perfusion units (PU). To observe microcirculation changing trend over time, PU within every five minutes were averaged. As shown in Figure 2, the measurement procedure was divided into the 5 min premoxibustion phase (baseline), moxibustion phases A, B, and C (segmented every 5 min of the 15 min moxibustion), and postmoxibustion phase (5 min after creasing moxibustion).

### 2.5. Statistical Analysis

Statistical analysis was performed using the statistical software package SPSS 22.0 for Windows (SPSS Inc., Chicago, IL, USA). The paired *t*-test was used to compare differences in fundamental physiological parameters between before and immediately after the LDF examination. PU data was expressed as mean ± standard error of mean (SEM) if they were normal distribution. PU change over time within and between measurement sites were analyzed by using a repeated measures analysis of variance (ANOVA). If significant differences were detected, Bonferroni post-hoc multiple comparison test was performed. A *p* value of less than 0.05 was considered statistically significant.

## 3. Results

All subjects completed the LDF examination and moxibustion intervention without any pain or discomfort. The fundamental physiological parameters in both groups are listed in Table 2. There were no significant changes in these parameters between before and immediately after the LDF examination (all *p* > 0.05) in both groups, indicating that the change of blood perfusion in the subjects was not influenced by physiological activities.

### 3.1. Blood Perfusion and Its Change Magnitude in Measurement Sites of Both Meridians in the Lung Meridian Intervention Group

**Figure 3:**
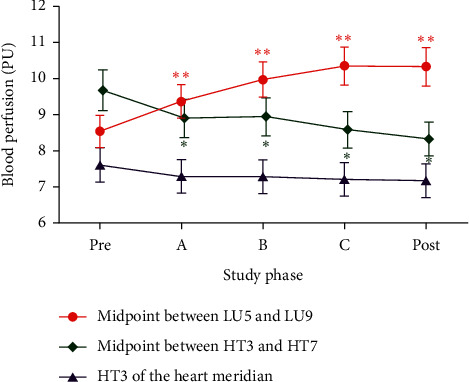
Changes of blood perfusion in three sites during different phases in the lung meridian intervention group. (i) Data are presented as mean ± SEM; (ii) “Pre” means 5 min premoxibustion phase, “A” means moxibustion phase A, “B” means moxibustion phase B, “C” means moxibustion phase C, “Post” means 5 min postmoxibustion phase; (iii) ^*∗∗*^*p* < 0.001 and ^*∗*^*p* < 0.01 when compared with premoxibustion PU.

In this group, moxibustion was applied at LU5 of the lung meridian. The blood perfusion and its corresponding change magnitude induced by LU5 moxibustion in measurement sites of both meridians are displayed in Figure 3 and Table 3. Following moxibustion at LU5 of the lung meridian, the PU of the distal site in the lung meridian (i.e., the midpoint between LU5 and LU9) increased significantly during moxibustion phase A, B, and C (all *p* < 0.001), with the change magnitude of 0.83 ± 0.15, 1.44 ± 0.21, and 1.81 ± 0.27, respectively, from premoxibustion PU. After the removal of moxibustion, the PU of the distal site in the lung meridian did not continuously increase, but within 5 min postmoxibustion phase, the microcirculation flux still kept at a higher degree, with the PU increased value of 1.78 ± 0.28 compared with premoxibustion. By contrast, the PU of HT3 in the heart meridian, which was the nearest to the moxibustion site, did not differ significantly during moxibustion phases A, B, and C and postmoxibustion phase (all *p* > 0.05). In addition, the PU in the distal site of the heart meridian (i.e., the midpoint between HT3 and HT7) tended to decrease gradually (all *p* < 0.01), with the decreased value of 0.77 ± 0.20, 0.73 ± 0.18, 1.09 ± 0.25, and 1.34 ± 0.24, respectively, when compared to premoxibustion PU.

Meanwhile, as shown in Table 3, a significant difference in the PU change from premoxibustion was detected between the distal site in the lung meridian (i.e., the midpoint between LU5 and LU9) and the other two control sites of the heart meridians during moxibustion phases A, B, and C and 5 min postmoxibustion phase (all *p* < 0.001).

### 3.2. Blood Perfusion and Its Change Magnitude in Measurement Sites of Both Meridians in the Heart Meridian Intervention Group

**Figure 4:**
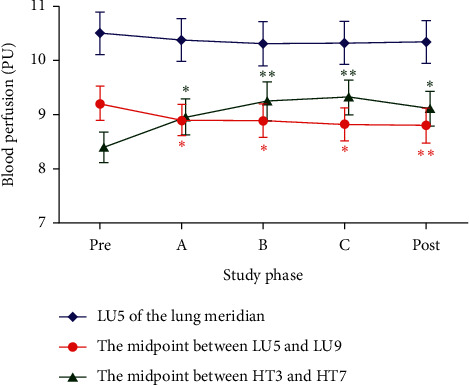
Changes of blood perfusion in three sites during different phases in the heart meridian intervention group. (i) Data are presented as mean ± SEM; (ii) “Pre” means 5 min premoxibustion phase, “A” means moxibustion phase A, “B” means moxibustion phase B, “C” means moxibustion phase C, “Post” means 5 min postmoxibustion phase; (iii) ^*∗∗*^*p* < 0.001 and ^*∗*^*p* < 0.01 when compared with premoxibustion PU.

In this group, moxibustion was applied at HT3 of the heart meridian. The blood perfusion and its corresponding change magnitude induced by moxibustion in measurement sites of both meridians are displayed in Figure 4 and Table 4. As it is shown, similar to the results of the lung meridian intervention group, the specificity of microcirculatory response between the heart and lung meridians was observed when moxibustion was performed in the heart meridian. In detail, following moxibustion at HT3, the PU in the distal site of the heart meridian (i.e., the midpoint between HT3 and HT7) increased significantly during moxibustion phases A, B, and C and postmoxibustion phase (all *p* < 0.01) when compared with premoxibustion. By contrast, as the nearest site from the moxibustion region, the PU of LU5 in the lung meridian did not change significantly at all measured time points when compared with premoxibustion (all *p* > 0.05). In addition, the PU in the distal site of the lung meridian (i.e., the midpoint between HT3 and HT7) declined significantly from baseline values at all measured time points during moxibustion and postmoxibustion (all *p* < 0.01). Meanwhile, as shown in Table 4, a significant difference in PU change was observed between the distal site of the heart meridian (i.e., the midpoint between HT3 and HT7) and the other two control sites of the lung meridian at all measured time points during moxibustion and postmoxibustion (all *p* < 0.001).

## 4. Discussion

### 4.1. Principal Findings

On one hand, the microcirculatory response of the lung meridian induced by moxibustion at LU5 was more significant than the heart meridian. Specifically, when LU5 of the lung meridian was stimulated by moxibustion, it could increase the blood perfusion in the distal site of the lung meridian significantly, whereas the blood perfusion of HT3 in the heart meridian, which was nearest to the moxibustion site, did not change significantly. Besides, blood perfusion in the distal site of the heart meridian tended to decrease gradually. After ceasing moxibustion, these microcirculatory responses at three measurement sites still persisted for 5 min, indicating that the effect after moxibustion could persist for 5 min, but the duration of after-effect needs further investigation by extending assessment time after the removal of moxibustion.

On the other hand, similar to moxibustion in the lung meridian, the specificity of microcirculatory response between the heart and lung meridians was observed when moxibustion was performed at HT3 of the heart meridian. These similar reactions in both groups indicated that moxibustion-induced microcirculatory responses may be more related to longitudinal-directional meridian routes (e.g., from LU5 to the midpoint between HT3 and HT7) than lateral-directional distance between two sites (e.g., from LU5 to HT3) when moxibustion is performed in these two meridians, respectively. Thus, the specificity for site-to-site association on the body surface between these two meridians was supported by the present study.

In addition, although previous studies demonstrated that acupoints are relatively silent under physiological conditions and turn relatively sensitive under pathological conditions [22], it is regarded that acupuncture tends to exert its effect under pathological conditions rather than physiological conditions [23]. Nevertheless, our findings revealed that meridians/acupoints are not only sensitized in pathological conditions, and their potential energy has existed and could also be activated by moxibustion in physiological state.

### 4.2. Highlights and Strengths

First, the majority of previous meridian studies in the past few years aimed to investigate the acupoint specificity and the correlation between body surface (i.e., meridians or acupoints) and internal organs. Nevertheless, there is a lack of studies designed to explore the correlation rules and specificity for site-to-site association on the body surface between different meridians, which is also a core scientific problem to be solved with respect to the practice of acupuncture, but it is often ignored. The investigation on specificity for site-to-site association between different meridians on the body surface can guide the acupoint selection in clinical acupuncture practice. For example, the poem of four command points is a classic summary guiding acupoint selection, which is also a typical representation of the specificity for site-to-site association between different meridians [19]. It is a classic example of distal treatment effect of acupoints along meridians and reflects the external specific communication between acupoints along meridians and the indications. In detail, according to the poem, acupuncture at Zusanli (ST 36) can treat diseases located in the abdomen; Weizhong (BL 40) can treat diseases located in the back; Lieque (LI 7) can treat diseases located in the head and napex; Hegu (LI 4) can treat diseases located in the face and mouth [19]. Nevertheless, the mechanisms underlying the specificity for site-to-site association on the body surface between different meridians remain inconclusive. Thus, more studies with rigorous designs are urgently needed to systematically investigate it. To the best of our knowledge, this is the first study to examine the specificity for site-to-site association on the body surface between two specific meridians systematically in humans by using LDF. The findings will in turn promote the research development on the specific effect of acupoints and optimize the clinical efficacy of acupuncture. Thus, more studies with rigorous designs are urgently needed to systematically investigate it. To the best of our knowledge, this is the first study to examine the specificity for site-to-site association on the body surface between two specific meridians systematically in humans by using LDF. The findings will in turn promote the research development on the specific effect of acupoints and optimize the clinical efficacy of acupuncture.

It is worth noting that the reason for selecting heart and lung meridians for comparison in our trial was due to the following considerations: (1) the circulation routes of these two meridians are shorter compared with other meridians; (2) the distribution of these two meridians on the body surface is adjacent in the arm; (3) the functions and indications of them are relatively simple and there are fewer visceral organs connected by them. These factors make the heart and lung meridians more suitable for investigating the specificity for site-to-site association on the body surface between two different meridians. Moreover, the lung is close to the heart anatomically, so these two viscera have close association physiopathologically. From clinical perspectives, heart and lung diseases have close correlations and interactions. In acupuncture clinical practice, acupoints of the heart meridians can be used for treating lung diseases. In turn, acupoints of the lung meridians can also be adopted for treating heart diseases. Thus, it is of great significance to choose these two meridians to investigate the specificity for site-to-site association between them, which will also help verify the significance of selecting appropriate acupoints to optimize clinical efficacy of acupuncture.

Second, the robustness of evidence from previous meridian studies was often weakened by subjective assessments. Thus, the present study adopted an objective tool, LDF, to investigate the specificity for site-to-site association between two specific meridians as well as its underlying microcirculatory mechanism. It is a well-established technique to monitor the microcirculation with advantages of noninvasiveness and real-time measurement capability [24, 25].

Animal and clinical studies have proved LDF is a promising tool to evaluate the effect of acupuncture or moxibustion on the microcirculation. For example, in rats, Zhang et al. [26] found that the blood perfusion of vessels and the stomach increased significantly after electroacupuncture (EA) or acupuncture was performed at “Zusanli” (ST 36), while the blood perfusion of the stomach in the blank control group decreased gradually. In healthy adults, Lu et al. [27] used LDF to investigate the difference in microcirculatory response between the hand's dorsum and palm induced by acupuncture. They found that blood flow change of the hand's dorsum and palm was not significantly different among the sham acupuncture, manual acupuncture, and EA. Kuo et al. [28] observed that after acupuncture stimulation was given at “Hoku” (LI4) and induced De-Qi sensation immediately, it would result in an acceleration of blood flow in the distal “Quchi” (LI11) acupoint along the same meridian. Hsiu et al. [29] used LDF to investigate differences in the microcirculatory responses evoked by moxibustion between “Hoku” (LI4) and two nearby nonacupoints and revealed that the flux around the moxibustion site increased significantly. In patients, Sprott et al. [30] observed an increase in flux over the painful tender points in fibromyalgia patients and proposed that the improvement in microcirculation of the tender points might alleviate the pain. Nevertheless, none of these past studies were designed to explore the specificity for site-to-site association on the body surface between two specific meridians by using LDF.

### 4.3. Clinical Implications

Meridians are regarded as the foundation upon which traditional acupuncture theory is built. Although a great amount of time and effort has been spent on their investigation over the past decades, a definitive conclusion regarding the physical structure of meridians remains difficult to establish [31]. Although previous studies revealed that meridians are anatomically associated with nerves, blood vessels, lymphatic vessels, neurovascular bundles, or other anatomical structures [32–35], meridians are not totally equal to nerves, blood vessels, or other known anatomical structures. As a connection, adjustment, and response system, it is more likely that the essence of meridians involves very complex physical structures, including some currently unknown structures [16]. Some studies found that meridians may be channels of multiple structures that transform and transmit materials, heat, energy, and messages [16, 36]. Moreover, the intensity of heat transmission along the meridian direction is more significant than that along the nonmeridian direction [37]. Acupuncture and moxibustion can also increase the blood perfusion and temperature in the distal site along meridians [28, 38]. Therefore, in our study the effect of moxibustion-induced heat transmission along the meridian route (e.g., longitudinally from LU5 to the midpoint between LU5 and LU9) was more significant than that along the nonmeridian direction (e.g., laterally from LU5 to HT3), which could explain why moxibustion at LU5 induced more significant microcirculatory effect in the distal site (i.e., the midpoint between LU5 and LU9) of the lung meridian than HT3, although HT3 was very close to the moxibustion site (LU5). Thus, our study indicated that moxibustion-induced microcirculatory responses may be more related to longitudinal-directional meridian routes than the specific lateral-directional distance between two sites located at different meridians.

In addition, the findings concerning the meridian specificity for site-to-site association of the heart and lung meridians on the body surface in our study could support the significance of acupoints selection to optimize the clinical efficacy of acupuncture. Considering that the moxibustion stimulation on the lung meridian induced more significant microcirculatory response along the lung meridian than the heart meridian and similar responses were observed but in the heart meridian when moxibustion was performed in the heart meridian, in pathological conditions, the therapeutic effect of acupuncture performed directly on the disease-affected meridian is likely to be superior to that performed on nonaffected meridians. This is consistent with the results of a recent high-quality trial involving 404 patients of chronic stable angina, which revealed that acupuncture on the disease-affected meridian showed superior benefits in alleviating angina than acupuncture on the nonaffected meridian [39]. Other studies also revealed that stimulating the acupoints on the affected meridians could produce specific effects on regulating the corresponding organs, and acupoint specificity is the important basis for this regulation effect [40, 41]. However, the acupoint specificity on the affected meridians continues to be a conflicting theoretical problem in acupuncture field [23, 42]. In recent years, a large number of researches on acupoint specificity have been launched, but the results remain mixed and inconclusive. More systematic researches are needed.

### 4.4. Limitations

Several limitations have to be addressed. First, the sample size was small. However, as a pilot trial, the results of this study could provide evidence for the feasibility of this trial design as well as basic data for a full-scale trial in the future. Second, only two meridians and limited measurement sites of them were compared because a three-channel LDF was used, which has reduced the robustness regarding the specificity for site-to-site association on the body surface between different meridians. In our future study, we will seek other techniques, such as laser Doppler imaging and laser speckle imaging, to investigate more meridians and measure more sites. We will also first determine the best anatomic representative to investigate the phenomenon of propagated sensation along meridians based on more abundant data. Third, given that a longer LDF assessment period might reduce participant compliance because some participants are unable to completely avoid movement of the forearm for a very long period, only 25 min LDF assessment was performed, which included only 5 min postmoxibustion phase. Thus, the duration of moxibustion-induced after-effect on microcirculation needs further investigation.

## 5. Conclusions

For the heart and lung meridians, the effect of moxibustion-induced microcirculatory response may be more related to meridian routes than the specific distance between different sites. Thus, the specificity for site-to-site association on the body surface between these two meridians might exist. Nevertheless, given that only two meridians and limited sites of them were measured and compared, all current findings are not sufficiently robust. More systematic research should be conducted to investigate more meridians and more measurement sites in the future.

## Figures and Tables

**Table 1:** Anatomical location of acupoints and LDF measurement sites of the heart and lung meridians.

Acupoints and LDF measurement sites	Anatomical location
“Shenmen” (HT7)	On the anteromedial aspect of the wrist, radial to the flexor carpi ulnaris tendon, on the palmar wrist crease.
“Taiyuan” (LU9)	On the anterolateral aspect of the wrist, between the radial styloid process and the scaphoid bone, in the depression ulnar to the abductor pollicis longus tendon.
“Chize” (LU5)	On the anterior aspect of the elbow, at the cubital crease, in the depression lateral to the biceps brachii tendon.
“Shaohai” (HT3)	On the anteromedial aspect of the elbow, just anterior to the medial epicondyle of the humerus, at the same level as the cubital crease.
The midpoint of the lung meridian along the forearm	The midpoint between LU5 and LU9
The midpoint of the heart meridian along the forearm	The midpoint between HT3 and HT7

**Table 2:** Fundamental physiological parameters of the study subjects prior to and immediately after LDF examination in both groups (mean ± standard deviation).

Physiological parameters	Lung meridian intervention group		Heart meridian intervention group	
	Prior to LDF examination	Immediately after LDF examination	Prior to LDF examination	Immediately after LDF examination
SBP (mmHg)	115.65 ± 4.29	114.32 ± 4.37	110.98 ± 5.87	109.25 ± 8.87
DBP (mmHg)	76.75 ± 5.13	75.05 ± 4.30	70.13 ± 5.20	69.85 ± 6.58
HR (bpm)	77.45 ± 4.77	76.53 ± 4.57	71.05 ± 6.28	70.01 ± 6.52
Body temperature (°C)	36.59 ± 0.15	36.56 ± 0.14	36.44 ± 0.10	36.40 ± 0.16

SBP: systolic blood pressure; DBP: diastolic blood pressure; HR: heart rate; bpm: beats per minute.

**Table 3:** Blood perfusion and its corresponding change magnitude in both meridians in the lung meridian intervention group (mean ± SEM, PU).

	Premoxibustion phase	During 15 min moxibustion			5 min postmoxibustion phase
		Phase A	Phase B	Phase C
The midpoint between LU5 and LU9	8.54 ± 0.44	9.37 ± 0.47^*∗∗*^	9.98 ± 0.50^*∗∗*^	10.35 ± 0.52^*∗∗*^	10.33 ± 0.53^*∗∗*^
	—	(0.83 ± 0.15)^††##^	(1.44 ± 0.21)^††##^	(1.81 ± 0.27)^††##^	(1.78 ± 0.28)^††##^
The midpoint between HT3 and HT7	9.68 ± 0.56	8.91 ± 0.52^*∗*^	8.95 ± 0.52^*∗*^	8.59 ± 0.49^*∗*^	8.34 ± 0.47^*∗*^
	—	(−0.77 ± 0.20)	(−0.73 ± 0.18)	(−1.09 ± 0.25)	(−1.34 ± 0.24)
HT3 of the heart meridian	7.62 ± 0.47	7.30 ± 0.46	7.28 ± 0.45	7.21 ± 0.46	7.18 ± 0.45
	—	(−0.31 ± 0.14)	(−0.33 ± 0.16)	(−0.40 ± 0.18)	(−0.44 ± 0.19)

(i) PU: perfusion units. The values in parentheses were the increase (or decrease) values of PU compared with premoxibustion; (ii) ^*∗∗*^*p* < 0.001 and ^*∗*^*p* < 0.01, compared with premoxibustion PU; (iii) ^††^*p* < 0.001, compared with the midpoint between HT3 and HT7; ^##^*p* < 0.001, compared with HT3.

**Table 4:** Blood perfusion and its corresponding change magnitude in measurement sites of both meridians in the heart meridian intervention group (mean ± SEM, PU).

	Premoxibustion phase	During 15 min moxibustion			5 min postmoxibustion phase
		Phase A	Phase B	Phase C	
The midpoint between HT3 and HT7	8.39 ± 0.28	8.95 ± 0.33^*∗*^	9.24 ± 0.36^*∗∗*^	9.31 ± 0.33^*∗∗*^	9.11 ± 0.33^*∗*^
	—	(0.56 ± 0.15)^††##^	(0.85 ± 0.16)^††##^	(0.92 ± 0.16)^††##^	(0.71 ± 0.17)^††##^
LU5 of the lung meridian	10.49 ± 0.39	10.37 ± 0.39	10.30 ± 0.41	10.31 ± 0.40	10.33 ± 0.39
	—	(−0.12 ± 0.04)	(−0.19 ± 0.07)	(−0.18 ± 0.07)	(−0.16 ± 0.04)
The midpoint between LU5 and LU9	9.20 ± 0.32	8.89 ± 0.29^*∗*^	8.88 ± 0.31^*∗*^	8.82 ± 0.30^*∗*^	8.80 ± 0.33^*∗∗*^
	—	(−0.31 ± 0.08)	(−0.32 ± 0.09)	(−0.39 ± 0.09)	(−0.41 ± 0.07)

(i) PU: perfusion units. The values in parentheses were the increase (or decrease) values of PU compared to premoxibustion; (ii) ^*∗∗*^*p* < 0.001 and ^*∗*^*p* < 0.01, compared with premoxibustion PU; (iii) ^††^*p* < 0.001, compared with LU5; ^##^*p* < 0.001, compared with the midpoint between LU5 and LU9.

## Data Availability

All data are available upon reasonable request to the corresponding author.

## Supplementary Materials

Supplementary Materials STROBE Statement: checklist of items that should be included in reports.
